# Phosphodiesterase 5 inhibitor sildenafil in patients with heart failure with preserved ejection fraction and combined pre- and postcapillary pulmonary hypertension: a randomized open-label pilot study

**DOI:** 10.1186/s12872-020-01671-2

**Published:** 2020-09-10

**Authors:** Evgeny Belyavskiy, Artem Ovchinnikov, Alexandra Potekhina, Fail Ageev, Frank Edelmann

**Affiliations:** 1 Department of Internal Medicine and Cardiology, Charité – Universitätsmedizin Berlin, Campus Virchow - Klinikum, Augustenburger Platz 1, 13353 Berlin, Germany; 2 DZHK (German Centre for Cardiovascular Research), partner site Berlin, Berlin, Germany; 3 Out-Patient Department, Institute of Clinical Cardiology, National Medical Research Center of Cardiology of the Ministry of Health of the Russian Federation, Moscow, Russia

**Keywords:** Pulmonary hypertension, Heart failure with preserved ejection fraction, Diastolic stress test, Diastolic dysfunction, Sildenafil

## Abstract

**Background:**

Heart failure with preserved ejection fraction (HFpEF) is frequently complicated by pulmonary hypertension (PH). A pulmonary vascular contribution could be considered as a substantial therapeutic target in HFpEF and PH and combined pre- and postcapillary PH (Cpc-PH).

**Methods:**

We enrolled 50 patients with HFpEF and Cpc-PH who were determined by echocardiography to have pulmonary artery systolic pressure (PASP) > 40 mmHg, pulmonary vascular resistance > 3 Wood units, and/or transpulmonary gradient > 15 mmHg.

**Results:**

The patients were assigned to the phosphodiesterase 5 (PDE5) inhibitor sildenafil group (25 mg TID for 3 months followed by 50 mg TID for 3 months; *n* = 30) or the control group (*n* = 20). In the sildenafil group after 6 months, the 6-min walk distance increased by 50 m (95% CI, 36 to 64 m); substantial improvement in NYHA functional class and exercise capacity during diastolic stress test were revealed; decreases in early mitral inflow to mitral annulus relaxation velocities ratio by 2.4 (95% CI, − 3.3 to − 1.4) and PASP by 17.0 mmHg (95% CI, 20.4 to 13.5) were observed; right ventricular systolic function (M-mode tricuspid annular plane systolic excursion) increased by 0.42 cm (95% CI, 0.32 to 0.52 cm; *P* < 0.01 for all). No changes occurred in the control group.

**Conclusions:**

In a subset of patients with HFpEF and Cpc-PH assessed by echocardiography, PDE5 inhibition was associated with an improvement in exercise capacity, pulmonary haemodynamic parameters, and right ventricular function. The role of sildenafil needs to be considered in randomized trials in selected patients with HFpEF with invasively confirmed Cpc-PH.

**Trial registration:**

Russian National Information System of Research, Development and Technology Data of Civilian Usage (NIS, https://rosrid.ru), registration number 01201257849. Registered 20 April 2012.

This manuscript adheres to the CONSORT guidelines.

## Background

Approximately one-half of patients with heart failure (HF) have a preserved ejection fraction (HFpEF) [1]. Risk factors of HFpEF **i**nclude age, hypertension, coronary heart disease, obesity, and diabetes [2]. At least 50% of patients with HFpEF develop pulmonary hypertension (PH) [3–5]. The development of HFpEF is related to an increase in left atrial (LA) pressure. With increased severity and duration of LA pressure overload pulmonary vascular disease (PVD) can develop by an increase in pulmonary arterial tone and/or intrinsic arterial remodeling. Haemodynamically these progressive pathologic alterations of the pulmonary arterial vasculature manifest by a rise in the pulmonary vascular resistance (PVR) and the condition is defined as combined pre- and postcapillary pulmonary hypertension (Cpc-PH) [6, 7]. As a result, the nonmuscular right ventricle is typically incapable of matching its contractile performance to the increasing afterload [8]. Compared with isolated postcapillary PH (Ipc-PH), Cpc-PH is commonly associated with right ventricular failure and worse prognosis [9]. Patients with Cpc-PH are younger than patients with Ipc-PH, despite similar comorbidities and prevalence, severity, and chronicity of left heart disease, and display genes and biological pathways in the lung known to contribute to “pulmonary arterial hypertension” pathophysiology [10].

There is growing evidence that phosphodiesterase 5 (PDE5) inhibition safely targets the above- mentioned alterations of the pulmonary arterial vessels, thus unloading the RV in left-sided PH [11] PDE5 inhibitors also possess beneficial pleiotropic left ventricular (LV) diastolic effects in HFpEF [12]. However, two trials failed to corroborate this finding in patients with HFpEF and PH [13, 14] Both trials investigated the effects of PDE5 inhibition predominantly in patients with Ipc-PH. Thus, the role of PDE5 inhibition in patients with HFpEF and pulmonary vasculopathy remains to be evaluated. We aimed to investigate the effect of chronic PDE5 inhibition with sildenafil on exercise capacity, RV function, and pulmonary haemodynamic parameters in patients with HFpEF and Cpc-PH determined by echocardiography.

## Methods

### Study population

The present randomised, controlled, open single-centre study took place over 6 months and was performed in the Out-Patient Department of the National Medical Research Center of Cardiology in Moscow (Russian Federation). We enrolled 50 patients with stable heart failure of New York Heart Association (NYHA) functional class II-III with preserved LV ejection fraction (> 50%) and Cpc-PH determined by echocardiography as high LV-filling pressures (LV diastolic dysfunction grade II/III) [15] and pulmonary artery systolic pressure (PASP) > 40 mmHg. The pre-capillary pulmonary component was determined by PVR > 3 Wood units and/or transpulmonary gradient (TPG) > 15 mmHg [16].

The exclusion criteria included the receipt of nitrates, advanced pulmonary disease or alternative causes of PH, revascularization within 3 months, evidence of myocardial ischemia during stress echocardiography, chronic atrial flutter/fibrillation, significant left-sided structural valve disease, hypertrophic cardiomyopathy, infiltrative or inflammatory myocardial diseases, pericardial disease, severe or very severe chronic obstructive pulmonary disease (GOLD stage III-IV), or noncardiac conditions precluding participation. Informed consent was obtained from all individual participants included in the study, which was conducted in accordance with the Declaration of Helsinki. The study protocol was approved by the local Institutional Ethic Committee.

### Study design

The participants were randomly assigned in an open-label fashion to receive PDE5 inhibitor sildenafil (*n* = 30) or to a control group (*n* = 20) in a 3:2 ratio via an automated web-based system (randomized.com), and allocation concealment was guaranteed by sequentially numbered, opaque, sealed envelopes. After obtaining informed consent from each patient, the envelope was opened by an outside coworker. Patients received 25 mg of sildenafil thrice daily for the first 3 months with a further increase to 50 mg thrice daily for another 3 months. Both the investigators and the participants were informed of the treatment allocation. The first dose of sildenafil was administered immediately after randomization under the supervision of the investigators. If adverse effects developed, study staff could recommend discontinuation or return to a lower or previously tolerated dose of the study drug. Basic heart failure therapy had been stable for at least 3 months. Echocardiography, 6-min walk test (6MWT), exercise echocardiography (diastolic stress test), and N-terminal pro–brain natriuretic peptide (NT-proBNP) blood level analysis were performed at baseline and 6 months after randomization; echocardiography also was performed at 3 months after randomization.

### Echocardiography

Echocardiographic assessment (iE33, Philips ultrasound machine) was performed by two experienced cardiac sonographers blinded to the patient data and treatment. Ventricular dimensions, wall thickness, chamber volumes, and LV ejection fraction were determined using current recommendations [17]. LV diastolic function was assessed by measuring the mitral inflow velocities (E, A), averaged mitral annulus relaxation velocity (mitral e′), and mitral E/e′ ratio [15].

Right heart assessment included right ventricle (RV) size (RV basal diameter), systolic (pulsed Doppler peak velocity at the tricuspid annulus [s′], M-mode tricuspid annular plane systolic excursion [TAPSE]), and diastolic function (pulsed Doppler of tricuspid inflow and hepatic vein, tissue Doppler of lateral tricuspid annulus [e′ and E/e′ ratio], 2-dimensional measurements of RA volume), and PASP with the estimation of right atrial (RA) pressure based on inferior vena cava (IVC) size and collapse [18].

PVR was estimated by the ratio of peak tricuspid regurgitation velocity (TRV) and velocity-time integral of the RV outflow tract (RVOT_VTI_), and the eq. *0.16 + 10 × TRV/RVOT*_*VTI*_ was used as a reliable method to identify patients with elevated PVR [19]. Mean pulmonary artery pressure was derived from the time to peak velocity of the RV outflow velocity curve (acceleration time, AcT_RVOT_): *90–0.62 ×* AcT_RVOT_ [20]. The eq. *1.25 × mitral Е/e′ + 1.90* was used to estimate the mean pulmonary capillary wedge pressure (PCWP) [21]. TPG was calculated as the pressure difference between the estimated mean pulmonary artery pressure and PCWP. All measurements represent the mean of ≥3 beats.

### Diastolic stress test

Patients exercised supine bicycle ergometry at 60 rpm starting with a 3-min period of low-level 25-W workload followed by 10-W increments in 1-min stages to maximum tolerated levels. During the test, two-dimensional images, mitral inflow velocities, mitral annulus tissue Doppler velocities, and TRV by continuous-wave Doppler were analysed at baseline, at the peak, and during recovery. Because of the peak TRV may vary with respiration during exercise and to minimize the measurement error, we used the average value between multiple beats (5–7 heart cycles), rather than the maximum.

### NT-proBNP

Plasma NT-proBNP level was measured via automated electrochemiluminescence immunoassay (Roche Diagnostics, Germany). The detection limit of the NTproBNP assay was 5 pg/mL.

### Study end points

The primary endpoint of this trial was the change in 6-min walking distance after 6 months of therapy. Secondary objectives included the change in NYHA functional class, exercise duration and maximal achieved workload during cycle ergometry, mitral E/e*′* ratio and PASP both at rest and during diastolic stress after 6 months of therapy. Using other prespecified endpoints, we also assessed the effect of PDE-5 inhibition on echo-estimated left and right ventricular structure and function, and NT-proBNP.

### Statistical analysis

The change in PASP was used to estimate the sample size needed to achieve adequate statistical power for the current study. We assumed a 44% decrease in systolic pulmonary artery pressure (≈24 mmHg) after 6 months of treatment in the study by Guazzi M. and colleagues [22]. In this study, 22 patients with HFpEF and left-sided PH demonstrated PASP of 54.5 mmHg with a standard deviation of 6.3 mmHg. Thus, at an α of 0.05 (two-sided) and a σ of 6.3, a sample size of 20 patients per group was required to achieve a power of 90%.

Normally distributed data are presented as mean ± standard deviation; nonnormally distributed data are presented as median (interquartile range). Categorical variables are reported as the number and percentages of observations. For normally distributed data, one-way analysis of variance was applied to the change from baseline, and for nonnormally distributed data, the Wilcoxon test was applied. The differences in parameters at baseline and after treatment between groups were tested using a Student *t*-test for normally distributed data and Mann–Whitney *U*-test for nonnormally distributed data. The treatment effects are presented using point estimates and 95% confidence intervals (CIs). The correlation between continuously distributed variables was tested by univariate regression analysis. A value of *P* < 0.05 was considered statistically significant. Statistical analysis was performed using standard software (MedCalc, version 17.1).

## Results

### Patient baseline characteristics and compliance

**Fig. 1:**
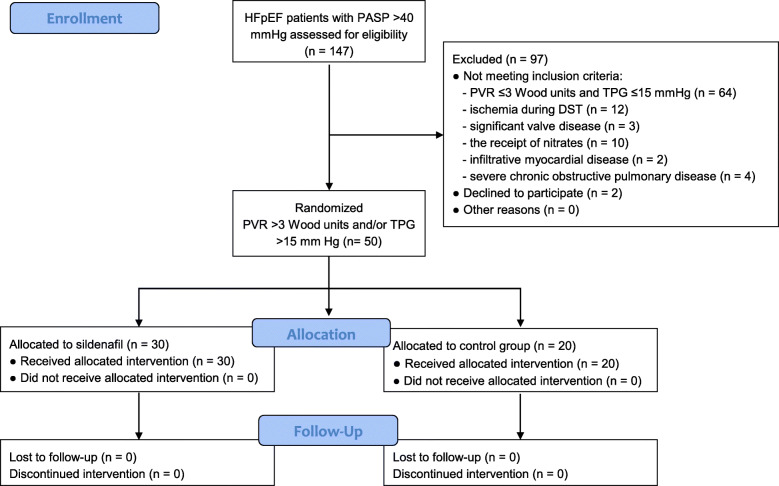
Flow chart of patient enrollment. DST, diastolic stress test; HFpEF, heart failure with preserved ejection fraction; PASP, pulmonary artery systolic pressure; PVR, pulmonary vascular resistance; TPG, transpulmonary gradient

A total of 147 subjects with HFpEF and PH were screened in the period between January 2013 and March 2014 (Fig. 1). Fifty patients met inclusion/exclusion criteria and were included in the final cohort; 30 received sildenafil in daily dosages prespecified by the study protocol: 75 mg for 3 months followed by 150 mg for another 3 months. The mean age of the patients was 71 years, and 52% were women. Study subjects were mainly obese with multiple comorbidities including long-standing hypertension (70% with concentric LV hypertrophy), ischemic heart disease, diabetes, and chronic kidney disease (Table 1).

**Table 1:** Baseline demographic and clinical characteristics and cardiovascular parameters in sildenafil and control group

	Sildenafil (*n* = 30)	Control (*n* = 20)	Total study population (*n* = 50)
Age, y	71 ± 7	71 ± 8	71 ± 7
Men, n (%)	17 (57)	7 (35)	24 (48)
NYHA II/III, n (%)	20/10 (67/33)	13/7 (65/35)	33/17 (66/34)
Systolic blood pressure, mm Hg	130 ± 14	127 ± 12	129 ± 13
Diastolic blood pressure, mm Hg	80 ± 11	76 ± 10	78 ± 11
Heart rate, bpm	63 ± 8	63 ± 7	63 ± 7
Body mass index, kg/m^2^	30 ± 6	29 ± 4	30 ± 5
Overweight/obesity (body mass index ≥25 kg/m^2^), n (%)	26 (87)	18 (90)	44 (88)
Hypertension (blood pressure ≥ 140/90 mmHg), n (%)	30 (100)	20 (100)	50 (100)
Paroxysmal atrial fibrillation, n (%)	10 (33)	5 (25)	15 (30)
Ischemic heart disease, n (%)	15 (50)	7 (35)	22 (44)
Previous myocardial infarction, n (%)	9 (30)	4 (20)	13 (26)
Myocardial revascularization, n (%)	11 (37)	4 (20)	15 (30)
Diabetes mellitus, n (%)	10 (33)	4 (20)	14 (28)
Chronic kidney disease, n (%)	26 (87)	14 (70)	40 (80)
Drug therapy:			
ACEI/ARB, n (%)	30 (100)	20 (100)	50 (100)
β-Blockers, n (%)	21 (70)	18 (90)	39 (78)
Diuretics, n (%)	28 (93)	19 (95)	47 (94)
Thiazide diuretics, n (%)	2 (7)	2 (10)	4 (8)
Loop diuretics, n (%)	26 (87)	17 (85)	34 (86)
Spironolactone, n (%)	6 (20)	2 (10)	8 (16)
Calcium channel blockers, n (%)	15 (50)	7 (35)	22 (44)
Statins, n (%)	26 (87)	18 (90)	44 (88)
LV ejection fraction, %	60 ± 5	61 ± 6	61 ± 5
LV hypertrophy, n (%)	20 (67)	15 (75)	35 (70)
PA systolic pressure, mm Hg	58.6 ± 14.9	55.5 ± 13.5	57.3 ± 14.3
Increased RA pressure, n (%)	26 (87)	14 (70)	40 (80)
RV systolic dysfunction, n (%)	22 (73)	10 (50)	32 (64)
PVR, Wood units	3.33 ± 0.64	3.19 ± 0.47	3.27 ± 0.58
TPG, mm Hg	23.3 ± 7.7	22.1 ± 8.8	22.8 ± 8.1
Mitral Е/e′ ratio	14.2 ± 4.2	12.6 ± 3.7	13.6 ± 4.0
LV diastolic dysfunction, grade II/III, n (%)	19/11 (64/36)	14/6 (70/30)	33/17 (66/34)
NT-proBNP, pg/mL	391 (190—582)	468 (205—720)	408 (194—631)

*NYHA* New York Heart Association, *ACEI* Angiotensin-converting enzyme inhibitor, *ARB* Angiotensin receptor blocker, *LV* Left ventricular, *PA* Pulmonary artery, *RA* Right atrial, *RV* Right ventricular, *PVR* Pulmonary vascular resistance, *TPG* Transpulmonary gradient, *Е* Early inflow velocity, *e′* annulus relaxation velocity, *NT-proBNP* N-terminal pro–brain natriuretic peptide

NS for all parameters

Most patients had echocardiographic signs of RV dysfunction: baseline elevated mean RA pressure was revealed in 80% and RV systolic dysfunction (TAPSE < 1.7 cm and/or tricuspid s′ < 9.5 cm/s) [17] in 64%. The groups were comparable in demographic and haemodynamic characteristics and current medical treatment (Table 1).

No patient from either group was lost to follow-up. Sildenafil was well tolerated by all patients; there was no symptomatic hypotension, facial flushing, or vision changes. Systemic blood pressure and heart rate did not vary significantly from the baseline values in either group (Table 2). Two patients in the control group required diuretic potentiation because of paroxysmal nocturnal dyspnoea.

**Table 2:** Effect of sildenafil on clinical and echocardiographic parameters

Variables	Sildenafil		Control	
	Baseline	6 months	Baseline	6 months
Systolic arterial pressure, mm Hg	130 ± 14	133 ± 16	127 ± 12	132 ± 17
Diastolic arterial pressure, mm Hg	80 ± 11	79 ± 8	76 ± 10	79 ± 10
Heart rate, bpm	63 ± 8	62 ± 8	63 ± 7	62 ± 6
Pulmonary vascular resistance, Wood units	3.33 ± 0.64	2.69 ± 0.49^**§§^	3.19 ± 0.47	3.16 ± 0.45
Acceleration time of RVOT, ms	83 ± 17	114 ± 24^**§§^	87 ± 17	88 ± 14
Pulmonary artery systolic pressure, mm Hg	58.6 ± 14.9	41.6 ± 10.3^**§§^	55.5 ± 13.5	56.4 ± 12.4
Transpulmonary gradient, mm Hg	23.3 ± 7.7	12.7 ± 9.1^**§§^	22.1 ± 8.8	21.0 ± 7.1
LV end diastolic dimension, cm	5.30 ± 0.47	5.37 ± 0.51^*^	5.14 ± 0.46	5.15 ± 0.42
Interventricular septum, cm	1.20 ± 0.10	1.14 ± 0.08^**§§^	1.20 ± 0.09	1.20 ± 0.09
LV posterior wall, cm	1.20 ± 0.10	1.14 ± 0.08^**§§^	1.20 ± 0.09	1.20 ± 0.09
LV mass index, g/m^2^	135 ± 52	111 ± 40^**^	130 ± 44	131 ± 47
LA volume index, mL/m^2^	50.7 ± 7.4	44.5 ± 6.4^**§§^	49.8 ± 8.5	50.2 ± 8.4
Mitral e′, cm/s	6.3 ± 1.4	7.3 ± 1.5^**^	6.7 ± 1.6	6.6 ± 1.4
Mitral E, cm/s	86 ± 19	83 ± 16	81 ± 16	83 ± 16
Mitral E/e′ ratio	14.2 ± 4.2	11.8 ± 3.2^**^	12.6 ± 3.7	13.2 ± 3.8
RV basal diameter, cm	4.80 ± 0.55	4.50 ± 0.52^**^	4.69 ± 0.56	4.68 ± 0.54
RA volume index, mL/m^2^	45.4 ± 6.5	38.5 ± 6.3^**^	46.6 ± 8.8	46.8 ± 8.6
Tricuspid e′, cm/s	8.2 ± 2.8	11.4 ± 2.6^**§§^	8.9 ± 2.9	8.4 ± 2.5
Tricuspid Е/e′ ratio	7.2 ± 3.4^§^	4.0 ± 1.4^**§^	5.3 ± 1.8	5.9 ± 2.2
IVC size, cm	2.26 ± 0.33	1.89 ± 0.37^**§^	2.08 ± 0.31	2.13 ± 0.35
IVC collapse with a sniff, %	34 ± 8^§^	53 ± 13^**§§^	42 ± 14	42 ± 13
TAPSE, cm	1.76 ± 0.38	2.18 ± 0.47^**§^	1.88 ± 0.37	1.91 ± 0.37
TAPSE/PASP, mm/mm Hg	0.32 ± 0.09	0.55 ± 0.17^**§§^	0.36 ± 0.13	0.36 ± 0.12
s′_RV_, cm/s	9.6 ± 2.1	12.2 ± 3.2^**§^	10.6 ± 2.0	10.7 ± 1.9
NT-proBNP, pg/mL	391 (190–582)	416 (227–671)	468 (205–720)	470 (195–697)

*OT* outflow tract, *LA* left atrial, *IVC* inferior vena cava, *TAPSE* tricuspid annular plane systolic excursion, *PASP* pulmonary artery systolic pressure, *s′*_*RV*_ peak velocity at the tricuspid annulus

^*^ — *P* < 0.05, ^**^ — *P* < 0.01 vs baseline

^§^— *P* < 0.05, ^§§^— *P* < 0.01 vs corresponding value in control group

### Primary endpoint

**Fig. 2:**
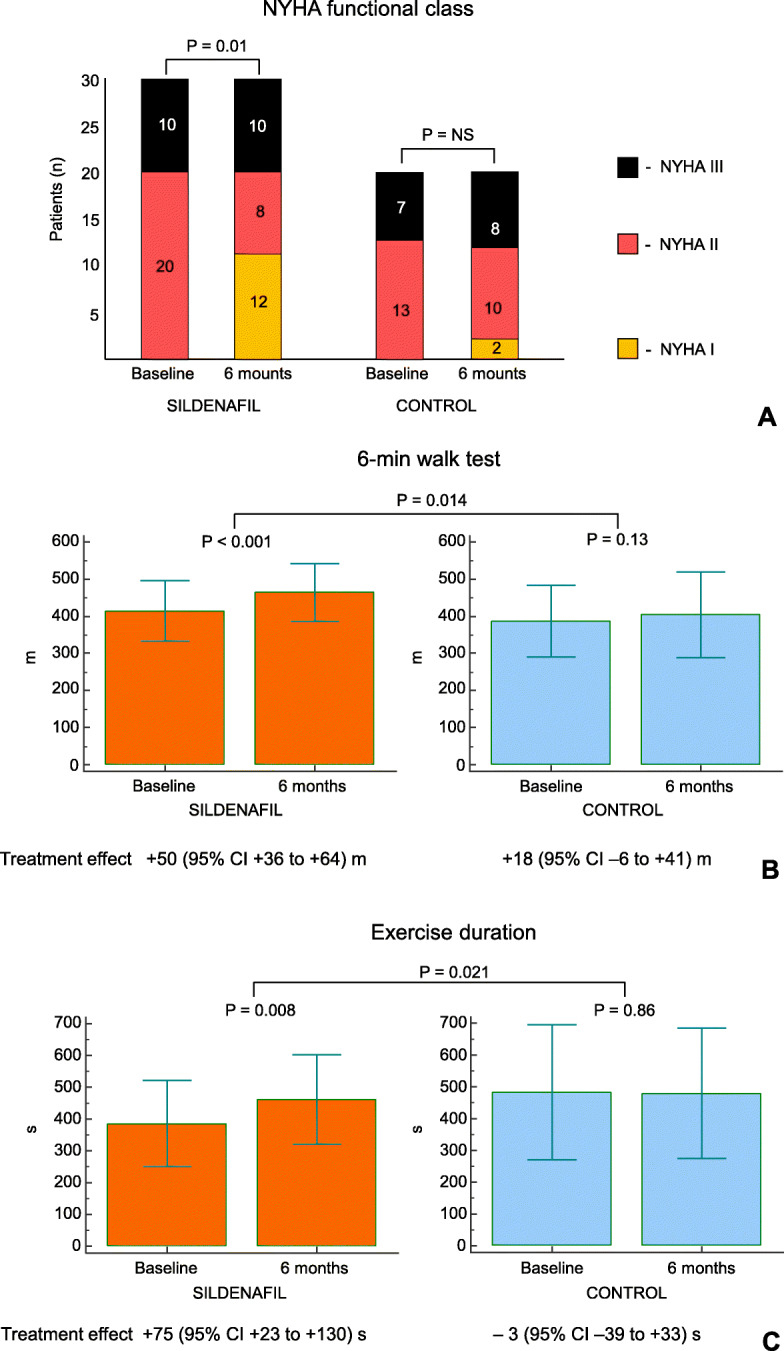
Six-minute walk distance (**a**), bicycle exercise duration (**b**), and NYHA functional class (**c**) at baseline and after 6 months in both study groups

After 6 months of therapy, an increase in 6-min walking distance by 50 m (95% CI, 36 to 64 m) was revealed in the sildenafil group (Fig. 2a); no significant changes occurred in the control group.

### Heart failure severity and functional capacity

In the sildenafil group after 6 months, exercise duration during the incremental bicycle test increased by 75 s (95% CI, 23 to 130 m; Fig. 2b), which was accompanied by substantial improvement in NYHA functional class (Fig. 2c). No changes occurred in the control group.

### Pulmonary and right heart haemodynamics

In the sildenafil group, the mean estimated PVR decreased from baseline values by 0.65 (95% CI, − 0.76 to − 0.53, *P* < 0.001) Wood units, and AcT_RVOT_ (strongly inversely correlating to PVR) increased by 31 (95% CI, 23 to 40, *P* < 0.001) ms. The improvements in PVR and AcT_RVOT_ were achieved within the first 3 months of low-dose therapy with sildenafil (75 mg per day; − 0.56 [95% CI, − 0.70 to − 0.42], *P* < 0.001] Wood units and + 29 [95% CI, 20 to 38, *P* < 0.001] ms, respectively), while a further 3 months of high-dose therapy (150 mg per day) provided a less prominent effect (− 0.09 [95% CI, − 0.20 to 0.02, NS] Wood units and + 2 [95% CI, − 5 to 10, NS] ms, respectively). No changes occurred in the control group over the same periods.

**Fig. 3:**
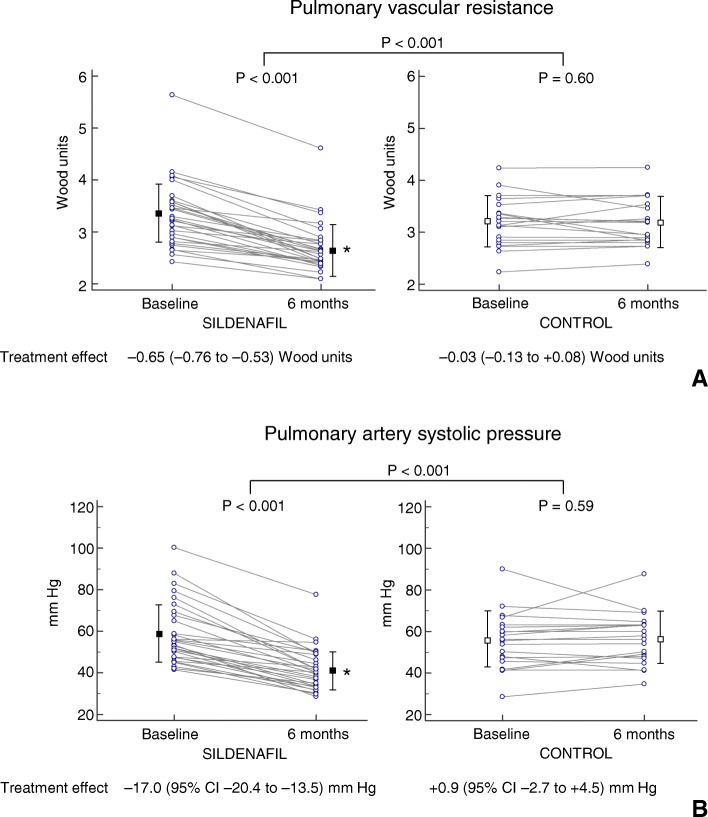
Individual and mean (± standard deviation) values of pulmonary vascular resistance (**a**) and pulmonary artery systolic pressure (**b**) at baseline and after 6 months of therapy

The PVR decline corresponded to a decrease in resting PASP by 17.0 (95% CI, 20.4 to 13.5, *P* < 0.001) mm Hg in the sildenafil group, whereas the average change in PASP in the control group was 0.9 (95% CI, − 2.7 to 4.5) mm Hg (*P* < 0.001 vs. sildenafil group, Table 2). We emphasize that a decrease in PVR and PASP after 6 months of therapy was observed in each patient in the sildenafil group (Fig. 3).

The decline in PVR was associated with RV size regress and systolic function improvement (RV basal dimension − 0.30 [95% CI, − 0.42 to − 0.18] cm, TAPSE + 0.42 [95% CI, 0.32 to 0.52] cm, tricuspid annulus s´ + 2.6 [95% CI, 1.8 to 3.4] cm/s) and TAPSE to PASP ratio (+ 0.24 [95% CI, 0.20 to 0.27] mm/mm Hg as an indicator of RV-arterial coupling) [23] (*P* < 0.001 for all).

Therapy with sildenafil was associated with RV diastolic function improvement and central venous pressure descent, as evidenced by a decrease in RA volume index (by 7.0 [95% CI, − 8.6 to − 5.3] mL/m^2^), tricuspid E/e′ ratio (by 3.2 [95% CI, − 4.2 to − 2.2]), and IVC size (by 0.37 [95% CI, − 0.49 to − 0.24] cm), an increase in IVC collapse with a sniff (by 19 [95% CI, 14 to 24]%), and tricuspid e′ velocity (by 3.2 [95% CI, 2.4 to 4.0] cm/s; *P* < 0.001 for all vs. baseline values in the sildenafil group and *P* < 0.001 vs. corresponding data in control group, Table 2). The right heart parameters remained unchanged in the control group during the study.

**Fig. 4:**
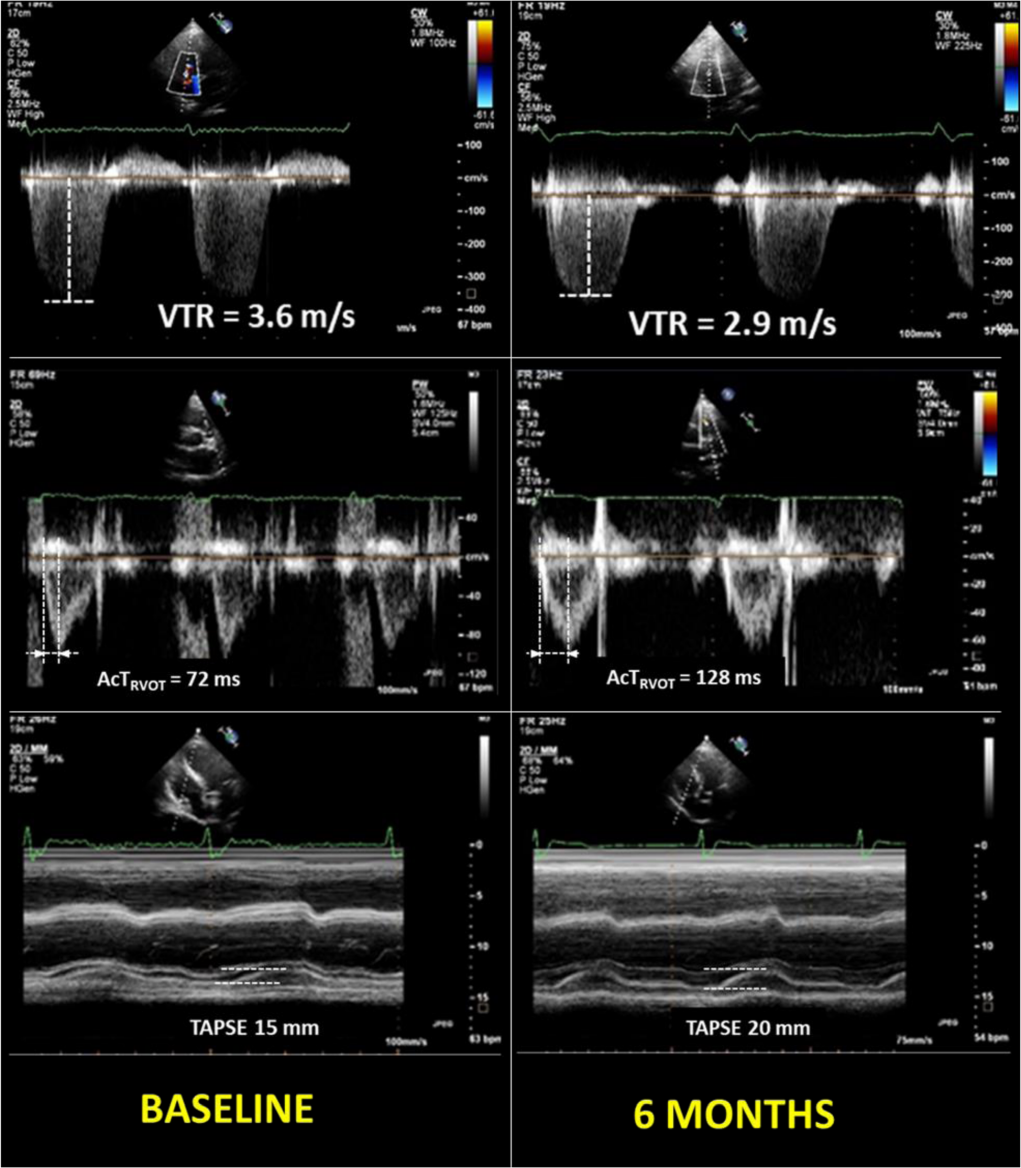
An example of Doppler peak tricuspid regurgitation and right ventricular outflow tracings, and tricuspid annular plane systolic excursion in a study patient with HFpEF at baseline (left panel) vs. after 6-month sildenafil therapy (right panel). Sildenafil therapy was associated with improvements in pulmonary artery systolic pressure (a decrease in tricuspid regurgitation velocity, TVR), pulmonary vascular resistance (an increase in the time to peak velocity of right ventricular outflow velocity, AcT_RVOT_), and right ventricular systolic function (an increase in tricuspid annular plane systolic excursion, TAPSE)

The change in estimated PVR in the total study population was inversely correlated with TAPSE (*r* = − 0.59) and tricuspid s′ velocity (*r* = − 0.48) dynamics but was directly correlated with RA volume index (*r* = 0.52) and tricuspid Е/e′ ratio dynamics (*r* = 0.46, *P* < 0.001 for all), supporting the pronounced relation between PVR decline and RV systolic and diastolic function improvement in patients with HFpEF. An example of the change in echo-derived pulmonary hemodynamics and right ventricular function in one study patient from the sildenafil group is shown in Fig. 4.

### LV structural and haemodynamic parameters

After 6 months of therapy, the mean change in mitral E/e′ ratio was − 2.4 (95% CI, − 3.3 to − 1.4; *P* < 0.001) in the sildenafil group and 0.6 (95% CI, 0 to 1.1; *P* = 0.05 vs. baseline and *P* < 0.0001 vs. sildenafil group) in the control group (Table 2). The improvement in PCWP (estimated by mitral E/e′ ratio) in the sildenafil group was accompanied by a significant reduction in LA volume index (− 6.2 [95% CI, − 7.7 to − 4.8] mL/m^2^) and LV mass index (− 24 [95% CI, − 34 to − 14] g/m^2^, both *P* < 0.001). LV mass index regress was correlated with PCWP decrease after 6 months of treatment (*r* = 0.37, *P* = 0.009). No significant changes in mitral e′ velocity, LA volume, or LV mass index occurred in the control group during 6 months (Table 2).

The prevailing mean pulmonary artery pressure (PAP) decline versus PCWP decline governed a significant decrease in TPG (− 10.5 [95% CI, − 14.1 to − 7.0] mm Hg; Table 2). The TPG in the sildenafil group achieved values that were almost twofold lower as compared with baseline, approaching the upper limit of the reference values.

Despite the decrease in PCWP in the sildenafil group, the plasma level of NT-proBNP remained unchanged in both groups (Table 2).

### Exercise haemodynamics

Sixteen patients (53%) in the sildenafil group and 12 (60%) in the control group performed supine bicycle exercise. At baseline, patients completed 59 ± 20 W in the sildenafil group and 76 ± 33 W in the control group (*P* = 0.11). Both exercise time and peak workload during diastolic stress test were increased after 6 months of therapy in the sildenafil group (+ 75 [95% CI, 23 to 130] s, *P* = 0.008 and + 11 [95% CI, 3 to 20] W, *P* = 0.013, respectively, vs. baseline) but not in the control group (− 3 [95% CI, 95% CI − 39 to 33] s and − 1 [95% CI, − 7 to 6] W, respectively). The increase in exercise duration after 6 months of therapy with sildenafil correlated with the improvement in resting RV systolic and diastolic function (TAPSE and tricuspid Е/e′ ratio: *r* = 0.43 and *r* = − 0.50, respectively; *P* < 0.05 for both) but not with changes in resting PCWP (mitral E/e′ ratio: *r* = − 0.09, *P* = 0.61), demonstrating the importance of RV function for exercise capacity in HFpEF patients with PH.

At baseline, the exercise was associated with a prominent increase in PASP (estimated by TRV) in both groups but with only a modest increase in PCWP (estimated by mitral E/e′ ratio). In five patients in the sildenafil group (17%) and in five patients in the control group (25%), the E/e′ ratio decreased during the peak exercise at baseline stress test.

**Fig. 5:**
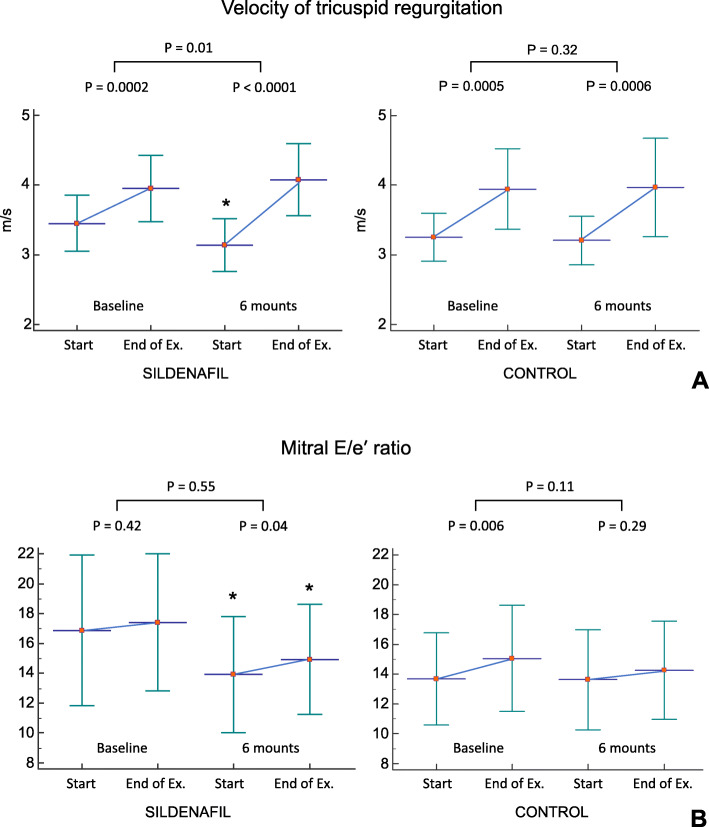
Changes in pulmonary artery systolic pressure (PASP, estimated by tricuspid regurgitation velocity, **a**) and in pulmonary capillary wedge pressure (PCWP, estimated by mitral E/e′ ratio, **b**) during cycle exercise at baseline and after 6 months. Ex., exercise. * *P* < 0.05 vs baseline

At the 6-month stress test in the sildenafil group, the resting TRV was lower, although exercise TRV elevation was greater as compared with the data at baseline stress test (Fig. 5). The same dynamics were observed for mitral E/e′ ratio: lower resting values but greater exercise elevation (Fig. 5). No changes in resting or peak exercise TRV or mitral E/e′ ratio were detected in the control group.

### The effects of different sildenafil dose regimens

The improvements in pulmonary haemodynamics (PVR, TPG, and AcT_RVOT_) were achieved within the first 3 months of low-dose therapy with sildenafil (75 mg per day) while the further 3 months of high-dose therapy (150 mg per day) provided less prominent effect (Table 3). In contrast, the progress in RV size and contractility (TAPSE) had been occurring gradually with significant improvements both after the low-dose and high-dose regimens. The similar gradual pattern was observed for PWCP (mitral E/e′ ratio) and LV mass, whereas the enhancement in LV relaxation (mitral e′ velocity) occurred after the low-dose therapy (Table 3).

**Table 3:** The effects of low vs high dose regimen of sildenafil on exercise capacity and echocardiographic variables

	Sildenafil		
	Low dose effect (Baseline – 3 months)	High-dose effect (3 months – 6 months)	Overall treatment effect
PVR, Wood units	−0.56 (95% CI − 0.70 to − 0.42)^**^	−0.09 (95% CI − 0.20 to 0.02)	−0.65 (95% CI − 0.76 to − 0.53)^**^
RVOT acceleration time, ms	29 (95% CI 20 to 38)^**^	2 (95% CI −5 to 10)	31 (95% CI 23 to 40)^**^
TAPSE, cm	0.25 (95% CI 0.18 to 0.31)^**^	0.17 (95% CI 0.10 to 0.25)^††^	0.42 (95% CI 0.32 to 0.52)^**^
RV basal diameter, cm	−0.18 (95% CI − 0.26 to − 0.10)^**^	−0.12 (95% CI − 0.21 to − 0.04)^††^	−0.30 (95% CI − 0.42 to − 0.18)^**^
Mitral e′, cm/s	0.8 (95% CI 0.5 to 1.0)^**^	0.2 (95% CI − 0.1 to 0.5)	1.0 (95% CI 0.6 to 1.3)^**^
Mitral E/e′ ratio	−1.2 (95% CI − 2.0 to − 0.5)^**^	−1.1 (95% CI − 1.8 to − 0.4)^††^	− 2.4 (95% CI − 3.3 to − 1.4)^**^
LV mass index, g/m^2^	−12 (95% CI − 21 to − 4)^**^	−12 (95% CI − 20 to − 4)^††^	−24 (95% CI − 34 to − 14)^**^

*Е* Early inflow velocity, *e′* Annulus relaxation velocity, *IVC* inferior vena cava, *LV* left ventricular, *OT* outflow tract, *PVR* Pulmonary vascular resistance, *RV* Right ventricular; *TAPSE* Tricuspid annular plane systolic excursion

^*^ — *P* < 0.05, ^**^ — *P* < 0.01 vs baseline

^†^ — *р* < 0.05, and ^††^ — *р* < 0.01 vs 3 month exam

## Discussion

In the present single-centre, randomised study, 6-month therapy with the PDE5 inhibitor sildenafil was associated with an increase in exercise capacity in patients with HFpEF and predominantly Cpc-PH determined by echocardiography. The beneficial effects observed were a decrease in PVR and improvement in RV systolic and diastolic function. Our data, therefore, support the use of sildenafil in these selected HFpEF patients.

PH is increasingly recognised as a significant complication of HFpEF, and some patients with left-sided PH might benefit from medical therapy previously considered to be suitable only for pulmonary arterial PH. PDE5 is selectively expressed in the pulmonary endothelial cells, and its inhibition by sildenafil leads to an increase in cyclic guanosine monophosphate (cGMP) level and protein kinase G activity. The potential benefits of PDE5 inhibition are posted to be due to the abundant PDE5 expression in the pulmonary vessels and to the unique properties of PDE5 inhibition selectively targeting pulmonary and intrapulmonary circulation rather than the systemic circulation [11]. Although PDE5 inhibition has numerous beneficial pleiotropic cardiovascular effects in HFpEF [12] the elimination of the reactive pulmonary component is presumed to be of predominant value in patients with HFpEF with concomitant Cpc-PH.

Clinical trials of sildenafil in HFpEF patients have demonstrated contradictory results. The early study by Guazzi M. and colleagues reported positive effects of sildenafil therapy on haemodynamics and RV function in HFpEF patients who predominantly fulfilled the hemodynamic criteria of Cpc-PH [22]. However, two subsequent larger trials failed to reveal any benefits and raised a question about the usefulness of sildenafil in HFpEF. In the RELAX trial [13], which involved 216 patients with HFpEF, therapy with sildenafil (20 mg TID for 12 weeks followed by 60 mg TID for 12 weeks) did not result in significant improvement in exercise capacity or clinical status as compared with placebo. Pulmonary haemodynamic or RV function assessment, however, were beyond the scope of the study, and PH was not a decisive entry criterion. More recently, Hoendermis ES and coworkers investigated 52 patients with PH due to HFpEF [14]. In contrast to RELAX, the participants were required to demonstrate invasively proven PH for eligibility. Patients were randomised to the PDE5 inhibitor sildenafil, titrated to 60 mg three times a day, or placebo for 12 weeks. The treatment with sildenafil neither reduced PAPs nor improved clinical parameters. Only 35% of patients in this trial developed precapillary PH (PVR > 3 Woods units), so the authors defined participants as having predominantly postcapillary PH. Thus, these neutral results do not clarify whether patients with HFpEF and Cpc-PH may benefit from PDE5 inhibition. Recently, a meta-analysis of randomised trials that compared PDE5 inhibition with placebo in chronic heart failure showed that effects of PDE5 inhibition in patients with HFpEF were heterogeneous, and the beneficial effect of PDE5 inhibition was related to the baseline PAP as well as the extent of PDE5 inhibition–mediated PAP decrease [24].

We selectively enrolled patients with PH associated with the pre-capillary pulmonary component determined by echocardiography. In HFpEF, if the PASP exceeds 50 mmHg, this may be indicative of additional pulmonary vascular disease as opposed to a pure consequence of left-sided HF [25]. The mean PASP was higher in our HFpEF group (57 mmHg) compared with both the RELAX trial (41 mmHg) and the study by Hoendermis ES et al. (52 mmHg), which supports the assumption of elevated PVR as a marker of the severity of pulmonary vasculopathy.

A data from the COMPERA registry showed an improvement in functional class, exercise capacity, and natriuretic peptides in 226 patients with PH and HFpEF receiving pulmonary vasodilators, predominantly PDE5 inhibitors [26]. These patients had very high TPG (on average 26 mmHg) and PVR (on average 7 Wood units), assuming pulmonary vascular disease and supporting the idea that the Cpc-PH phenotype may benefit from therapies targeting pulmonary circulation. More recently, Kramer T et al. showed in a retrospective study the beneficial effect of PDE5 inhibitors on 6-min walk distance, functional class, NT-proBNP levels, right ventricular function, and hospitalization rate in 40 hemodynamically precisely characterized patients with HFpEF and Cpc-PH [27].

In the current study, sildenafil substantially eliminated the reactive precapillary pulmonary component, as reflected by a decrease in the mean PVR and mean PASP. This indicates that pulmonary vasculopathy can be, at least partly, reversible and might be a primary therapeutic target. We emphasize that the improvements in pulmonary haemodynamics occurred in all patients receiving sildenafil (Fig. 3) and were mainly achieved during the first 3 months of the treatment, providing support for the hypothesis that the decrease in pulmonary arterial tone is a major component of the acute effects of PDE5 inhibition [28].

Therapy with sildenafil improved RV contractile and diastolic function, decreased RV size, and RA pressure (Table 2). There was an increase in TAPSE/PASP ratio determining the improvement in RV-arterial coupling, as TAPSE is a surrogate of contractile function and PASP generally reflects the afterload. A decreased TAPSE/PASP ratio emerged as the echocardiography-derived independent predictor of Cpc-PH [29] and a potent prognostic marker in heart failure [23].

These improvements were presumably caused by the effective elimination of high PVR, since there were correlations between the changes in estimated PVR and RV function. Our findings are in accordance with the results of Guazzi M. and colleagues [22] and Kramer T. and colleagues [27], suggesting a long-term sustained role of sildenafil in improving RV contractility and RA due to a reduction in PAP in patients with PH and HFpEF. The significant improvement in RV contractile function in our patients (TAPSE and tricuspid s′ velocity increase) could occur due to both RV afterload reduction and enhanced contractility. PDE5 is highly expressed in the hypertrophied human right ventricle, and PDE5 inhibition improves the contractility of failing RV cardiac myocytes [30].

In the present study, therapy with sildenafil was associated with a decrease in LV mass and improvement in LV diastolic dysfunction (Table 2). We suppose that the effect on LV diastolic dysfunction was due to direct lusitropic potency rather than a reduction in LV afterload, since the systemic arterial pressure was stable (Table 2). The reduction in LV mass index was correlated with PCWP decrease during therapy, considering the role of other effects besides the lusitropic effects of sildenafil (antihypertrophic, antifibrotic) [31]. In animals with pressure overload, PDE5 inhibition did not show antihypertrophic effects in mice with less severe pressure overload, whereas dramatic benefits were observed in mice with severe pressure overload, eccentric LV hypertrophy, and pulmonary congestion [32, 33]. The patients in our study demonstrated pronounced LV hypertrophy (mean LV mass index was 133 g/m^2^) that was noticeably higher than in two other studies (< 80 g/m^2^), which showed no LV diastolic benefits [13, 14]. Excessive LV remodeling associated with high PDE5 activation in patients with advanced HFpEF might preferentially benefit from PDE5 inhibition. Finally, RV and RA distension in patients with Cpc-HF adversely alters LV diastolic dysfunction [34] and sildenafil could modulate this by reducing right heart pressures and volumes.

Therapy with sildenafil was associated with a decrease in resting PASP but, paradoxically, with a significant increment in PASP during exercise (Fig. 5). Given that, PVR remains unchanged during exercise even in patients with severe PVD [35], the exercise-induced elevation of PASP most commonly arises from the interaction between an increased RV cardiac output and a rise in the LA pressure [36]. In patients with early HFpEF, the LV filling pressure increases significantly during exercise [37]. At baseline, we observed only a modest increase in PCWP during exercise in patients with advanced HFpEF. Moreover, in 10 patients (20%), the E/e′ ratio decreased during peak exercise. Butler and colleagues demonstrated a paradoxical decrease in PCWP during exercise in patients with heart failure and severe PH as a result of LV underfilling due to reductions in RV output [38]. In the present study, 6-month therapy with sildenafil was associated with a significant increment in PCWP during exercise (Fig. 5), which might reflect the restoration of LV preload. The greater peak exercise elevation in PCWP was associated, however, with lower resting and peak PCWP absolute values as compared with the baseline stress test values. Thus, we suppose that the LV filling pressure decreased after sildenafil therapy.

The significant increase in PASP response during exercise was probably due to the improvements in RV contractile reserve. A blunted RV contractile reserve is the main reason for exercise limitation in patients with HFpEF and PH [39]. In patients with stable pulmonary arterial hypertension, a rise in the peak TR jet velocity after exercise was associated with better exercise-induced RV function and better clinical outcome [40].

The abnormal LV diastolic filling pressure is the key functional abnormality in HFpEF and leads to release of cardiac natriuretic peptides [41]. Improvements in RV and LV filling pressures were not, however, accompanied by a decrease in NT-proBNP level in the present study. We speculate that the LV filling pressure, although decreased by sildenafil, remained elevated enough to stimulate the sufficient production of NT-proBNP, as the filling of the noncompliant ventricle depends on elevated filling pressures. Second, since the law of Laplace dictates that the LV wall stress is inversely proportional to the LV wall thickness and directly proportional to LV filling pressures, the lack of a decrease in NT-proBNP might be due to the maintenance of relatively high diastolic wall stress despite improvement in LV filling pressures, as a regress in LV wall thickness also occurred. Finally, the improvement in LV filling pressure could be counterbalanced by the increased LV preload due to PVR regression.

The absence of an increase in NT-proBNP level in the sildenafil group might be of even greater significance for excluding a detrimental effect of increased LV preload on LV filling pressures. Elevated natriuretic peptide levels do not predict the response to treatment among HFpEF patients, and treatment response may be greatest in patients with low natriuretic peptide level [42, 43]. The complex interplay between factors that govern the natriuretic peptide level such as LV concentric remodeling, obesity, advanced age, and diabetes mellitus may affect NT-proBNP level dynamics in a heterogeneous population of HFpEF patients [44].

### Study limitations

The absence of placebo control, the conductance as a single-centre study, non-blinded design, and relatively small number of participants are among the limitations of the present study. The major limitation is the absence of invasive assessment of pulmonary haemodynamics, which, according to current recommendations, is the reference method for quantification of PAP [7]. However, echocardiography is the first-line noninvasive tool that should be performed whenever PH is suspected [7]. Echocardiographic estimates of RV and pulmonary vascular function are feasible both at rest and during exercise, identify pathology with reasonable accuracy, and represent valid screening tools for the identification of PVD in routine clinical practice [45].

The increased PVR was assessed by ultrasound and one may argue on the actual PVD in our study population. The noninvasive evaluation of PVR as the TRV/RVOT_VTI_ ratio was shown to correlate well with a wide range of invasive PVR measurements in large groups of patients with different PH aetiologies and may help detect pulmonary vasculopathy [19, 46–48]. In addition to PVR, we also used PASP and AcT_RVOT_ for PH diagnosis and treatment effect assessment. The mean PASP has been validated in numerous studies as a reliable marker for invasively assessed pulmonary haemodynamics [49, 50]. AcT_RVOT_ estimation is a valid method for identifying patients with high PVR and is still the prerequisite for diagnosis of pre- vs. postcapillary PH [51].

The ultrasound parameters applied in the present study were appropriate. The mean PVR was 3.4 ± 0.4 Wood units, and the calculated TPG was 23 ± 8 mmHg accompanied by stereotypical changes in RV, reduced contractility, and/or an increase in central venous pressure that assume Cpc-PH. Defined Cpc-PH according to echo-derived rather than invasive-derived parameters is probably a reasonable option, but this approach needs to be validated.

## Conclusion

In the present study, sildenafil significantly improved the exercise capacity and NYHA functional class in patients with HFpEF and combined pre- and postcapillary PH determined by echocardiography. These improvements appeared to be the results of beneficial effects on pulmonary vascular tone, RV contractile function, and the reduction in PCWP (both at rest and during exercise). Our data, therefore, indicate that selected patients with HFpEF and combined pre- and postcapillary PH assessed by echocardiography may benefit from targeted therapy with PDE 5 inhibition with sildenafil. However, because of several limitations of the current study, the role of sildenafil needs to be considered in future randomized trials in selected patients with HFpEF with invasively confirmed Cpc-PH.

## Data Availability

The datasets generated during and/or analysed during the current study are available from the corresponding author on reasonable request.

## Abbreviations

AcT: Acceleration time
ACEI: Angiotensin-converting enzyme inhibitor
ARB: Angiotensin receptor blocker
cGMP: Cyclic guanosine monophosphate
CI: Confidence interval
Cpc-PH: Combined pre- and postcapillary pulmonary hypertension
DST: Diastolic stress test
Е: Peak early diastolic mitral inflow velocity
e′: Early diastolic mitral annular velocity
HFpEF: Heart failure with preserved ejection fraction
Ipc-PH: Isolated postcapillary pulmonary hypertension
IVC: Inferior vena cava
LA: Left atrial
LV: Left ventricular
6MWT: 6-min walk test
NS: Nonsignificant
NT-proBNP: N-terminal pro–brain natriuretic peptide
NYHA: New York heart association
OT: Outflow tract
PA: Pulmonary artery
PASP: Pulmonary artery systolic pressure
PCWP: Pulmonary capillary wedge pressure
PDE5: Phosphodiesterase 5 inhibitor
PH: Pulmonary hypertension
PVR: Pulmonary vascular resistance
RA: Right atrial
RV: Right ventricular
s′_RV_: Tricuspid annular peak systolic velocity
TAPSE: Tricuspid annular plane systolic excursion
TID: Three times a day
TPG: Transpulmonary gradient
TRV: Tricuspid regurgitation velocity
VTI: Velocity-time integral
